# Supplementary material for: Recent deterioration of coral reefs in the South China Sea due to multiple disturbances
Source: PeerJ. 2022 Jul 25;10:e13634. doi: 10.7717/peerj.13634 (PMC9332401; doi:10.7717/peerj.13634)
Supplement: Supplemental Information 1 [file peerj-10-13634-s001.docx]

Table S1 Longitude and latitude information of 44 coral reef monitoring sites at six regions.

| **Region** | **Site** | **Longitude** | **Latitude** |
| --- | --- | --- | --- |
| Bei Jiao | BJ1 | 111.5623 | 17.1043 |
|  | BJ2 | 111.5417 | 17.0838 |
|  | BJ3 | 111.5216 | 17.0733 |
|  | BJ4 | 111.4821 | 17.0566 |
|  | BJ5 | 111.4533 | 17.0834 |
|  | BJ6 | 111.4740 | 17.1012 |
|  | BJ7 | 111.5002 | 17.1122 |
|  | BJ8 | 111.5299 | 17.1182 |
| Yongxing Dao | YX1 | 112.3419 | 16.8421 |
|  | YX2 | 112.3322 | 16.8402 |
|  | YX3 | 112.3327 | 16.8294 |
| Yongle Atoll | QF | 111.6520 | 16.5780 |
|  | YY | 111.6800 | 16.5936 |
|  | SY1 | 111.7490 | 16.5300 |
|  | SY2 | 111.7760 | 16.5360 |
|  | JQ | 111.7601 | 16.4563 |
|  | LY1 | 111.5779 | 16.4876 |
|  | LY2 | 111.6007 | 16.4749 |
|  | LY3 | 111.6055 | 16.4360 |
|  | LY4 | 111.5833 | 16.4412 |
|  | LY5 | 111.5698 | 16.4544 |
| Yuzhuo Jiao | YZ1 | 111.9575 | 16.3234 |
|  | YZ2 | 111.9799 | 16.3501 |
|  | YZ3 | 112.0104 | 16.3618 |
|  | YZ4 | 112.0434 | 16.3644 |
|  | YZ5 | 112.0795 | 16.3565 |
|  | YZ6 | 112.0813 | 16.3286 |
|  | YZ7 | 112.0464 | 16.3341 |
|  | YZ8 | 112.0105 | 16.3264 |
| Panshi Yu | PS1 | 111.7672 | 16.0687 |
|  | PS2 | 111.7971 | 16.0743 |
|  | PS3 | 111.8282 | 16.0648 |
|  | PS4 | 111.8204 | 16.0488 |
|  | PS5 | 111.7903 | 16.0318 |
|  | PS6 | 111.7718 | 16.0322 |
|  | PS7 | 111.7510 | 16.0543 |
| Langhua Jiao | LH1 | 112.5936 | 16.0788 |
|  | LH2 | 112.5431 | 16.0759 |
|  | LH3 | 112.5012 | 16.0627 |
|  | LH4 | 112.4354 | 16.0283 |
|  | LH5 | 112.4691 | 16.0059 |
|  | LH6 | 112.5151 | 16.0138 |
|  | LH7 | 112.5573 | 16.0346 |
|  | LH8 | 112.6020 | 16.0500 |
